# Failure of combined antiretroviral therapy intensification with maraviroc and raltegravir in chronically HIV-1 infected patients to reduce the viral reservoir: the *IntensHIV* randomized trial

**DOI:** 10.1186/1742-6405-11-33

**Published:** 2014-10-07

**Authors:** Alain Lafeuillade, Assi Assi, Cécile Poggi, Caroline Bresson-Cuquemelle, Eric Jullian, Catherine Tamalet

**Affiliations:** Department of Infectious Diseases, Sainte Musse, General Hospital, 54 rue Henri Sainte Claire Deville, 83100 Toulon, France; Department of Virology, General Hospital, Toulon, France; Department of Immunology, General Hospital, Toulon, France; Department of Biology, General Hospital, Toulon, France; Department of Virology, University Hospital, Marseille, France

**Keywords:** HIV intensification, cART Intensification, HIV reservoirs, HIV DNA, HIV remission, Maraviroc, Raltegravir

## Abstract

**Background:**

Ongoing HIV-1 replication in lymphoid cells is one explanation of the persistence of HIV-1 reservoirs despite highly active antiretroviral therapy (cART). We tested the potential of cART intensification by Maraviroc plus Raltegravir to decrease proviral HIV-1 DNA levels in lymphoid cells during a randomized trial.

**Patients and methods:**

We randomly assigned for 48 weeks 22 patients to continue their current first line regimen of Truvada® plus Kaletra® or intensify it with Maraviroc and Raltegravir. The primary objective was to obtain a 50% decrease in proviral HIV-1 DNA levels in lymphoid cells with intensification. Blood samples were drawn at W-2, W0, W2, W4, W12, W24 and W48. Plasma viremia, cellular proviral DNA and cellular RNA, 2-LTR circles and lymphocytes subsets were assayed using validated methods. Patients in the intensified group underwent a gut biopsy at baseline and W48 to measure proviral DNA levels. Statistical analysis used parametric and non-parametric tests.

**Results:**

Ten patients in each arm completed the trial. The 2 populations were comparable at baseline. No change in the reservoir size was observed in the intensified arm compared to the control arm measured in peripheral blood mononuclear cells (PBMCs). No change in the reservoir size was observed in gut proviral DNA in the intensified arm. In this group, no increase in 2-LTR circles was observed as early as 2 weeks after intensification and no change was found in residual plasma RNA levels measured by the single copy assay. However, a decrease in CD8^+^ T cells activation was observed at 24 and 48 weeks, as well as in PBMCs HIV-1 RNA levels.

**Conclusion:**

We conclude that the intensification of a Protease Inhibitor regimen with Maraviroc and Raltegravir does not impact the blood proviral DNA reservoir of HIV but can decrease the cell-associated HIV RNA, the CD8 activation and has a possible impact on rectal proviral HIV DNA in some patients.

**Trial registration:**

ClinicalTrials.gov identifier number NCT00935480

## Introduction

With the advent of combined antiretroviral therapy (cART), HIV-1 replication can become undetectable in the plasma for years. However, HIV-1 persists in lymphoid reservoirs where residual low levels of viral replication can be found [1]. Consequently, cART intensification could reduce the residual HIV-1 replication and, hence, the HIV-1 reservoir. The aim of this trial was to test the efficacy of cART intensification in first line treated patients to decrease the proviral HIV-1 reservoir in circulating peripheral blood mononuclear cells (PBMCs). We used Maraviroc, a CCR5 inhibitor and Raltegravir, an integrase inhibitor for intensification, with the objective to get at least a 50% decrease in proviral DNA levels in PBMCs.

## Patients and methods

Twenty-two adult patients on first line therapy with Truvada® (1 pill/day) and Kaletra® 400/100 (2 pills twice daily) were randomly assigned to continued cART of add Maraviroc (150 mg twice a day) and Raltegravir (400 mg twice a day) to their regimen.

Inclusion criteria included to be on first line therapy with this regimen, having no contraindication to receive Maraviroc and Raltegravir, have a plasma viral load <20 copies/ml for at least 12 weeks, no contraindication for gut biopsies.

CD4^+^, CD8^+^ and CD8^+^CD38^+^ T cell subsets were measured by flow cytometry (BD Le Pont de Claix, France) using commercially available monoclonal antibodies (BD Le Pont de Claix, France).

HIV-1 RNA levels were measured in plasma by using the Amplicor Monitor® assay (Roche Diagnostic, Meylan, France). Patients with levels <20 copies/ml where tested with the single copy assay (SCA) as previously reported [2]. HIV-1 RNA titers were also measured in lymphoid cells with a previously described technique evaluating both un-spliced and multiply spliced RNA [3].

HIV-1 DNA levels were measured by using the Generic HIV® assay (Biocentric, Bandol, France) according to manufacturer’s instructions.

2-LTR quantification used a previously reported technique [4].

Drug levels were measured by LC-MS.

CCR5 tropism was determined on PBMC HIV DNA by sequencing according to the ANRS protocol [5] using the geno2pheno with a cutoff of 20 percent.

Lymphoid tissue was obtained by sigmoid colon biopsies at 30 cm from the anus and processed as previously reported [6].

Only patients included in the intensified arm of the study were proposed to get gut biopsies at baseline and W48.

Patients were analyzed in blood at W-2, W0, W2, W4, W12, W24 and W48.

The randomization between the 2 groups was done by using a computer-based method. It was performed by our 2 technicians for clinical trials who also stored the trial data locked in their office.

The primary objective was to detect at least a 50% decrease in PBMCs proviral DNA levels in the intensified group and a calculation of at least 8 patients needed in each arm was done.

The secondary objectives were to measure the evolution of lymphocytes subsets, plasma viral load and PBMCs HIV-1 RNA levels in the 2 groups.

Statistical analysis was done with the SPSS v20® software (IBM, Bois-Colombes, France) and used descriptive statistics as well as parametric and non-parametric tests.

The Ethics Committee and the French Agency for Drugs Security (ANSM) approved the protocol. All patients gave their written informed consent before inclusion and the randomization was performed between W-2 and W0.

The protocol number on ClinicalTrials.gov is NCT00935480.

## Results

**Figure 1:**
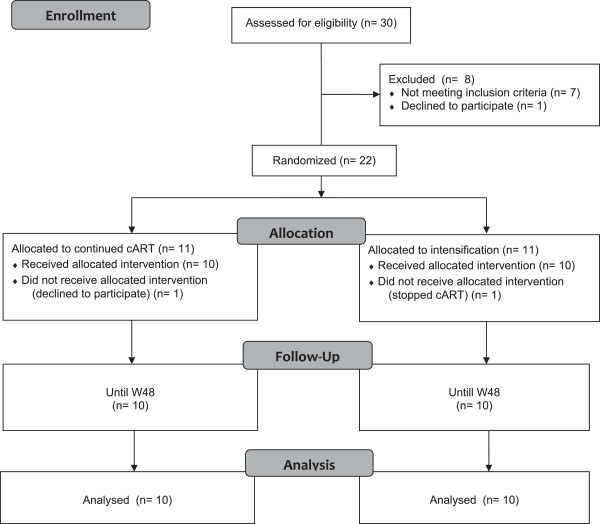
**Patients’ disposition during the trial.**

We screened a total of 30 patients and 10 were included in each arm (Figure 1).

All included patients were naïve of Maraviroc and Raltegravir and showed plasma HIV-1 RNA <20 copies/ml for a median duration of 24 weeks (range: 12–36). Baseline patients’ characteristics are shown in Table 1.

**Table 1:** **Baseline characteristics of patients**

	Arm 1 n = 10	Arm 2 n = 10
**Age** (Mean ± SD) years	54 ± 11	50 ± 12
**Sex** (M/F)	8/2	9/1
**Duration of HIV infection** (Mean ± SD) in months	20 ± 17	15 ± 3
**Duration of 1st line therapy** (Mean ± SD) in months	14 ± 12	10 ± 3
**Duration of undetectable viral load** (Mean ± SD) in monhs	13 ± 19	6 ± 2
**CD4** ^+^ **cells/mm** ^**3**^ (Mean ± SD)	598 ± 380	744 ± 377
**CD4** ^+^ **cells/mm** ^**3**^ **nadir** (Mean ± SD)	270 ± 120	290 ± 210
**CD8** ^+^ **cells/mm** ^**3**^ (Mean ± SD)	813 ± 414	927 ± 352
**Viral load single copy assay** Nb positive cases	1/10 (6 copies/ml)	1/10 (8 copies/ml)
**HIV DNA copies/10** ^**6**^ **PBMC** (Mean ± SD)	41 ± 37	24 ± 22
**HIV RNA copies/10** ^**6**^ **PBMC** (Mean ± SD)	43 ± 28	32 ± 23
**CCR5 tropism** Nb of patients	10/10	9/10

For each parameter, the data are pooled results obtained at W-2 and at W0 is used. SD: standard deviation.

Eleven patients were randomized to the continuing arm and 11 to the intensified arm.

In the continuing arm, 1 patient declined to follow the protocol beyond screening and baseline as he hoped to be in the intensified arm. In the intensified arm, 1 patient stopped antiretroviral therapy after week o and was excluded from the study.

Consequently, 20 patients were analyzed until week 48.

Both groups were comparable in terms of age, sex, duration of infection, CD4 cell count, HIV viremia, (SCA) HIV DNA levels, CCR5 tropism and markers of activation at baseline (Table 1).

Over the 48 weeks of follow-up, antiretrovirals were well tolerated in both groups and no adverse event was observed.

Adherence was correct in each group, assessed by residual through levels of lopinavir (group 1), lopinavir, Maraviroc and raltegravir (group 2). In each case, levels were in the needed range (data not shown).

Regarding the SCA, one patient in each group had detectable viremia at levels <10 copies/ml. These 2 cases did not show detectable viremia at any time point during the trial. On the contrary one patient negative at baseline in the control arm was found at 8 copies/ml at week 12 and 2 patients in the intensified arm were at 3 and 15 copies/ml respectively at weeks 12 and 24. These patients did not show any resistance selection at the time cART was initiated (data not shown).

Regarding 2-LTR, no patient was positive at baseline. No positive detection was found during the trial for all cases but one in the intensified group who was measured at 5.84 2-LTR units/10^6^ PBMC at week 2.

**Figure 2:**
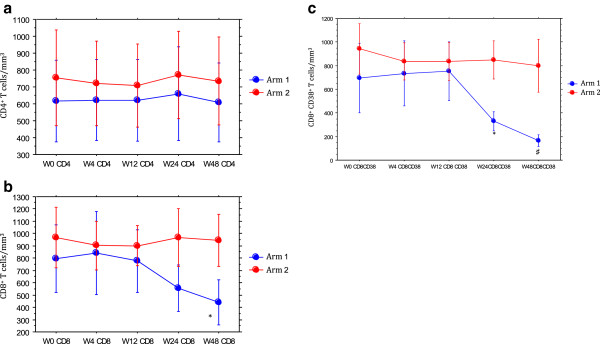
**Evolution in the 2 arms of immune markers over 48 weeks.** 2**a**: CD4^+^ T cells. 2**b**: CD8^+^ T cells. 3**c**: CD8^+^CD38^+^ T cells. Arm 1: intensified. Arm 2: controls. Results are shown as means with 95% confidence intervals (bars). * paired *t*-test: p = 0.01 compared to baseline. ♯ unpaired *t*-test: p = 0.002 compared to baseline.

The mean CD4^+^ T cell counts did not change over time in the 2 arms (Figure 2a).

The mean CD8^+^ T cell counts showed a statistically significant decrease in the intensified arm compared to baseline at weeks 24 and 48 (Figure 2b).

**Figure 3:**
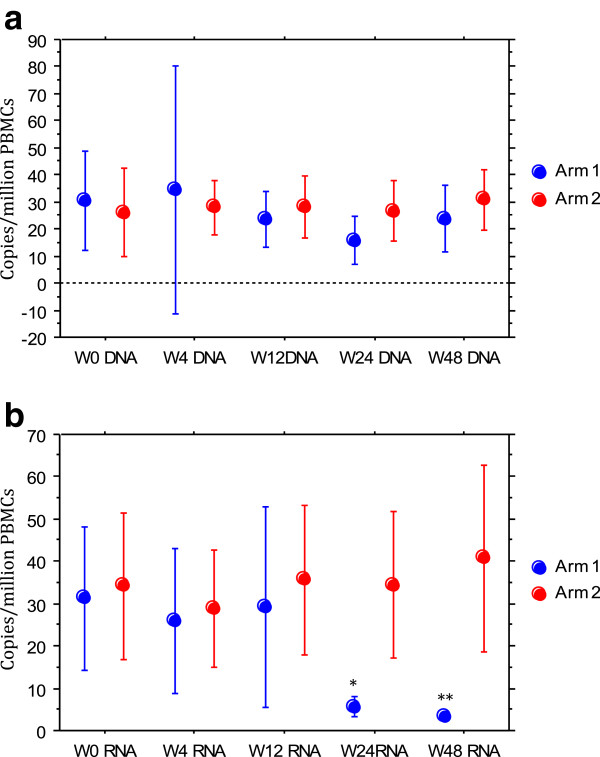
**Evolution in the 2 arms of PBMCs viral markers over 48 weeks in the 2 arms.** 3**a**: proviral HIV-1 DNA in PBMC. 3**b**: HIV-1 ARN in PBMCs. Arm 1: intensified. Arm 2: controls. Results are shown as means with 95% confidence intervals (bars). *non-parametric paired test: p = 0.07 compared to baseline; **p = 0.06 compared to baseline.

Regarding the CD8^+^CD38^+^ T cell counts, a statistically significant decrease in the intensified arm compared to baseline at weeks 24 and 48 was also found (Figure 2c).Proviral DNA levels in PBMCs did not change over time in the 2 arms (Figure 3a).

Mean HIV-1 RNA levels in PBMCs were similar in both groups at baseline (Table 1). A decrease was observed in the intensified arm at weeks 24 and 48 but not in the control arm. These results reached statistical significance (Figure 3b).

**Figure 4:**
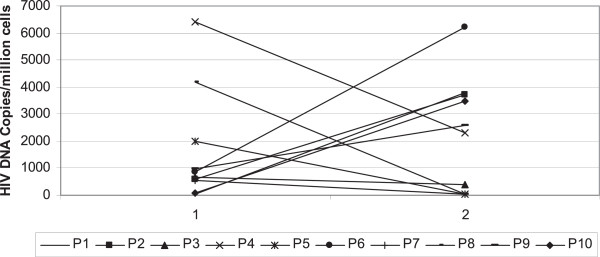
**Evolution of HIV-1 DNA in lymphoid cells taken from sigmoid colon in the intensified group (copies/10** ^**6**^ **cells).**

Regarding proviral DNA levels in gut cells, no trend was observed (Figure 4) despite the fact that we measured DNA levels in cells as a whole and not in sorted CD4^+^ T cells only [6, 7].

HIV-1 RNA levels in gut cells could not be assessed due to the lack of sufficient material left after measuring HIV-1 DNA.

## Discussion

Despite a nearly complete suppression of HIV-1 replication with cART, HIV-1 persists in lymphoid reservoirs [8] and plasma viremia always rebounds when therapy is stopped. These reservoirs are stable on cART even after years of compliance. Two mechanisms, which are not exclusive, have been proposed to explain this persistence: ongoing viral replication and/or homeostatic proliferation [9, 10].

By adding 2 new antiretroviral drugs with different mechanisms of action in first line Protease Inhibitor treated patients, we tried to impact the stability of the reservoir. The originality of this approach was the choice of drugs and the long period of intensification. However, no difference was found between intensified cases and the control group up to 48 weeks regarding the primary end point of the trial, which was a more than 50% decrease in proviral DNA levels in circulating lymphoid cells. Nevertheless we observed diverse patterns of proviral DNA evolution in gut cells but due to the small sample size we were unable to correlate these evolutions to any characteristic of the patient. No increase in 2-LTR levels, a marker of ongoing HIV-1 replication was demonstrated as early as 2 weeks after intensification. No change in CD4^+^ T cells was found but a late decrease of activation markers (CD8^+^CD38^+^) was observed in the intensified arm following a decrease of total CD8^+^ T cells. This decrease in activation markers might be due to the effect of intensification on ongoing viral replication. Interestingly, we also observed a decrease of PBMCs HIV-1 RNA levels in the intensified arm after 24 weeks that can also reflect the reduction of ongoing viral replication, as this marker has been demonstrated to reflect this replication [11].

Other trials have addressed the issue of cART intensification and showed conflicting results. In the study of Buzon et al., an increase in 2-LTR was demonstrated by adding Raltegravir [12]. In the trial reported by Dinoso et al., no effect of intensification was obtained on the residual viremia measured by the single copy assay [13].

To our knowledge, only two studies used Maraviroc and Raltegravir in combination [14, 15] and found negative results on the HIV reservoir.

Maraviroc has been shown to be an antiretroviral drug, but also to increase the expression of latent HIV [16]. It is impossible in our study to know if this effect was induced as Maraviroc was combined with Raltegravir.

Our study has some limitations. Firstly, as patients were effectively treated, CCR5 tropism could only be tested on archived virus. Secondly, the sample size was small due to the fact that having to perform gut biopsies discouraged patients. Thirdly, we were able only to measure the HIV-1 reservoir by PCR techniques and not by culture of replication competent virus [17]. However we observed a significant impact of intensification on blood cells ongoing HIV replication and markers of lymphocytes activation. Regarding the results on rectal proviral DNA, we cannot conclude due to the small sample size and no control group for this test. Nevertheless, previous studies have shown that rectal biopsies might not be the best representative of gut HIV reservoirs, compared to other locations like the ileum [18]. One other possible confounding factor is the fact that we included HIV naïve patients with a short time of undetectable viremia on cART. This could have explained the persistence of ongoing viral replication.

Further studies are needed with larger samples to confirm that ongoing viral replication is present in some patients despite effective therapy and can be reduced by drug intensification. This is a major question to resolve before moving to therapeutic trials aimed at inducing a HIV functional cure [19, 20].

## Conclusion

In the population studied, 48 weeks cART intensification with Maraviroc and Raltegravir failed to decrease the proviral HIV reservoir in circulating lymphocytes but was able to decrease both lymphocytes activation and ongoing viral replication. A possible effect on proviral DNA in rectal cells was observed in some patients but difficult to interpret due to the low sample size.

## References

[1] Svicher V, Ceccherini-Silberstein F, Antinori A, Aquaro S, Perno CF (2014). Understanding HIV compartments and reservoirs. Curr HIV/AIDS Rep.

[2] Palmer S, Wiegand AP, Maldarelli F, Bazmi H, Mican JM, Polis M, Bazmi H, Mican JM, Polis M, Dewar RL, Planta A, Liu S, Metcalf JA, Mellors JW, Coffin JM (2003). New real-time reverse transcriptase-initiated PCR assay with single-copy sensitivity for human immunodeficiency virus type 1 RNA in plasma. J Clin Microbiol.

[3] Fischer M, Gunthard HF, Opravil M, Joos B, Huber W, Bisset LR, Ott P, Böni J, Weber R, Cone RW (2000). Residual HIV-RNA levels persist for Up to 2.5 years in peripheral blood mononuclear cells of patients on potent antiretroviral therapy. AIDS Res and Human Retroviruses.

[4] Luo R, Cardozo EF, Piovoso MJ, Wu H, Buzon MJ, Martinez-Picado J, Zurakowski R (2013). Modelling HIV-1 2-LTR dynamics following raltegravir intensification. J R Soc Interface.

[5] Delobel P, Nugeyre MT, Cazabat M, Pasquier C, Marchou B, Massip P, Barre-Sinoussi F, Israël N, Izopet J (2007). Population-based sequencing of the V3 region of env for predicting the coreceptor usage of human immunodeficiency virus type 1 quasispecies. J Clin Microbiol.

[6] Lafeuillade A, Cheret A, Hittinger G, Bernardini D, Cuquemelle C, Jullian E, Poggi C (2009). Rectal cell-associated HIV-1 RNA: a new marker ready for the clinic. HIV Clin Trials.

[7] Yukl SA, Sinclair E, Somsouk M, Hunt PW, Epling L, Killian M, Girling V, Li P, Havlir DV, Deeks SG, Wong JK, Hatano H (2014). A comparison of methods for measuring rectal HIV levels suggests that HIV DNA resides in cells other than CD4+ T cells, including myeloid cells. AIDS.

[8] Eisele E, Siliciano RF (2012). Redefining the viral reservoirs that prevent HIV-1 eradication. Immunity.

[9] Sigal A, Kim JT, Balazs AB, Dekel E, Mayo A, Milo R, Baltimore D (2011). Cell-to-cell spread of HIV permits ongoing replication despite antiretroviral therapy. Nature.

[10] Chomont N, El-Far M, Ancuta P, Trautmann L, Procopio FA, Yassine-Diab B, Boucher G, Boulassel MR, Ghattas G, Brenchley JM, Schacker TW, Hill BJ, Douek DC, Routy JP, Haddad EK, Sékaly RP (2009). HIV reservoir size and persistence are driven by T cell survival and homeostatic proliferation. Nat Med.

[11] Pasternak AO, Lukashov VV, Berkhout B (2013). Cell-associated HIV RNA: a biomarker of viral persistence. Retrovirology.

[12] Buzón MJ, Massanella M, Llibre JM, Esteve A, Dahl V, Puertas MC, Gatell JM, Domingo P, Paredes R, Sharkey M, Palmer S, Stevenson M, Clotet B, Blanco J, Martinez-Picado J (2010). HIV-1 replication and immune dynamics are affected by raltegravir intensification of HAART-suppressed subjects. Nat Med.

[13] Dinoso JB, Kim SY, Wiegand AM, Palmer SE, Gange SJ, Cranmer L, O'Shea A, Callender M, Spivak A, Brennan T, Kearney MF, Proschan MA, Mican JM, Rehm CA, Coffin JM, Mellors JW, Siliciano RF, Maldarelli F (2009). Treatment intensification does not reduce residual HIV-1 viremia in patients on highly active antiretroviral therapy. Proc Natl Acad Sci U S A.

[14] Puertas MC, Massanella M, Llibre JM, Ballestero M, Buzon MJ, Ouchi D, Esteve A, Boix J, Manzardo C, Miró JM, Gatell JM, Clotet B, Blanco J, Martinez-Picado J, MaraviBoost Collaborative Group (2014). Intensification of a raltegravir-based regimen with maraviroc in early HIV-1 infection. AIDS.

[15] Achenbach C, Deeks S, Wilkin T, Berzins B, Casazza J, Lambert S, Assoumou L, Katlama C, Autran A, Murphy R, the ERAMUNE 02 Study Group (2014). Impact of RAL/MVC Intensification With or Without HIV-rAd5 Vaccination on HIV DNA: EraMune 02. Proceedings of the Conference on Retroviruses and Opportunistic Infections, Boston.

[16] Madrid-Elena N, Moreno S, Hernández-Novoa B, Solomon A, Gutiérrez C, García-Bermejo L, Solomon A, Lewin S (2013). The Effect of Maraviroc on HIV Transcription in Resting CD4+ T-cells from ART-suppressed HIV-1-infected Patients. Global Antiviral Journal.

[17] Ho YC, Shan L, Hosmane NN, Wang J, Laskey SB, Rosenbloom DI, Lai J, Blankson JN, Siliciano JD, Siliciano RF (2013). Replication-competent noninduced proviruses in the latent reservoir increase barrier to HIV-1 cure. Cell.

[18] Yukl S, Shergill A, McQuaid K, Gianella S, Lampiris H, Hare CB, Pandori M, Sinclair E, Günthard HF, Fischer M, Wong JK, Havlir DV (2013). Effect of raltegravir-containing intensification on HIV burden and T cell activation in multiple Gut sites of HIV + adults on suppressive antiretroviral therapy. AIDS.

[19] International AIDS Society Working Group on HIV Cure (2012). Towards an HIV cure: a global scientific strategy. Nat Rev Immunol.

[20] Hill AL, Rosenbloom DI, Fu F, Nowak MA, Siliciano RF (2014). Predicting the outcomes of treatment to eradicate the latent reservoir for HIV-1. Proc Natl Acad Sci U S A.

